# Potential mechanisms within the digital stress-management intervention *StressProffen* for cancer survivors: a qualitative study post randomized controlled trial

**DOI:** 10.3389/fpsyg.2025.1703540

**Published:** 2026-01-12

**Authors:** Cecilie Varsi, Elin Børøsund, Shawna L. Ehlers, Matthew M. Clark, Michael A. Andrykowski, Lise Solberg Nes

**Affiliations:** 1 Department of Digital Health Research, Division of Medicine, Oslo University Hospital, Oslo, Norway; 2 Faculty of Health and Social Sciences, University of South-Eastern Norway, Drammen, Norway; 3 Department of Nursing and Health Sciences, Faculty of Health and Social Sciences, University of South-Eastern Norway, Drammen, Norway; 4 Department of Psychiatry and Psychology, College of Medicine and Science, Mayo Clinic, Rochester, MN, United States; 5 Department of Behavioral Science, College of Medicine, University of Kentucky, Lexington, KY, United States; 6 Institute of Clinical Medicine, Faculty of Medicine, University of Oslo, Oslo, Norway

**Keywords:** cancer, stress-management, cognitive behavioral therapy, digital, health, intervention, qualitative

## Abstract

**Background:**

Cancer diagnoses and treatment are characterized by physical and psychological stressors. Evidence-based psychosocial stress-management interventions offer physical and psychological relief, but are not always available. Results from clinical trials show the digital, evidence-based, and user-centered stress-management intervention *StressProffen* to be associated with reduced stress, anxiety, and depression, as well as improved self-regulatory capacity and quality of life for cancer survivors. While such interventions can be efficacious, the actual mechanisms of these effects remain understudied.

**Objective:**

To qualitatively explore potential mechanisms in terms of psychological and behavioral change for cancer survivors after 12 months of access to *StressProffen*.

**Methods:**

Using a selection matrix to ensure variation in sex, age, diagnosis, and program progress, the current study invited 39 participants who had access to *StressProffen* over 12 months through a randomized controlled trial (RCT) to participate in post-RCT semi-structured qualitative interviews related to use and potential benefit. Interview transcripts were analyzed using thematic analysis.

**Results:**

Twenty-six cancer survivors completing the RCT agreed to participate. Qualitative analyses yielded five themes based upon patient-reported changes attributed to *StressProffen* use: (1) *Self-awareness and personal values;* who I am and what I want; (2) *Comprehension;* reflection and understanding of difficult thoughts and emotions; (3) *Social relationships;* manage my relations, (4) *Relaxation and focusing skills;* learn new relaxation, distraction, and focusing techniques; and (5) *Coping skills and adjustment;* coping with, adjusting to, and being prepared for difficult situations in all aspects of life.

**Conclusion:**

Access to digital stress-management interventions, such as *StressProffen*, has been shown to contribute to improved psychological wellbeing and quality of life for cancer survivors. Qualitative analyses of patient-reported benefits suggest mechanisms of enhanced self-awareness, improved comprehension of the relationship between thoughts and emotions, stronger social relationships, and improved skill self-efficacy in terms of relaxation, focusing, and coping skills. A mixed methods approach, including quantitative as well as qualitative analyses, can facilitate in-depth explorations of the impact of digital psychosocial interventions for cancer survivors.

## Background

Being diagnosed with and undergoing cancer treatment is often accompanied by an array of physical (e.g., fatigue, pain, and discomfort) and psychological challenges (e.g., stress, distress, anxiety, and depression) (Antoni and Dhabhar, 2019; Andersen et al., 1994; Grassi et al., 2017; Singer, 2018; Stanton et al., 2015; Stanton, 2006; Stanton, 2012), with an accompanying reduction in quality of life (QoL) (Antoni and Dhabhar, 2019; Andersen et al., 1994; Grassi et al., 2017; Singer, 2018; Stanton, 2006; Stanton, 2012; Clark et al., 2013; Solberg Nes et al., 2012).

While in-person psychosocial interventions for cancer stress management have demonstrated significant efficacy, positively impacting psychological wellbeing and quality of life (Antoni et al., 2023; Andersen et al., 2023; Stanton, 2006; Clark et al., 2013; Grassi et al., 2017; Andersen et al., 2004; Andrykowski and Manne, 2006; Antoni et al., 2001; Gudenkauf and Ehlers, 2018; Stagl et al., 2015), access to these evidence-based interventions is limited. Existing barriers to access include availability (i.e., such interventions are often only offered at larger, metropolitan academic medical centers), geographical distance to treatment site, limited insurance coverage, and cancer survivors feeling overwhelmed and/or unable to engage in individual or group treatments (Grassi et al., 2017).

To address access barriers, a new wave of digital health solutions is being developed. While demonstrating initial promise, the efficacy of digital self-management interventions in terms of psychological outcomes in cancer survivors has been variable (Willems et al., 2020; Escriva Boulley et al., 2018; Buneviciene et al., 2021; Seiler et al., 2017; Penedo et al., 2020; Fournier et al., 2023; Zhang et al., 2025). This variability highlights necessary directions for future digital solutions, such as (1) Being built upon a theoretical foundation, (2) Engaging stakeholders during both design and development, (3) Recruiting sufficient sample sizes, (4) Using validated outcome measures, and (5) Follow-up over an extended period of time (McAlpine et al., 2015; Seiler et al., 2017; Penedo et al., 2020; Lee et al., 2023; Fournier et al., 2023).

Responding to these identified gaps in digital health research, the current multidisciplinary research team developed *StressProffen*, a digital stress-management intervention for cancer survivors built upon the theoretical foundation of the cognitive behavioral therapy framework (CBT; Beck, 2011; Beck, 2020). *StressProffen* was designed and developed utilizing a user-centered design, in close collaboration between cancer survivors, researchers, and clinicians specialized within medical oncology, psychosocial oncology, and digital health experts (Borosund et al., 2018). A subsequent feasibility pilot study was conducted in line with recommendations for evaluating complex medical interventions (Skivington et al., 2021), demonstrating good acceptability, usability, and feasibility (Borosund et al., 2020b). Following intervention optimization based on feedback from the feasibility pilot study participants, *StressProffen* was subsequently tested in a randomized controlled trial (RCT) with cancer survivors (*n* = 172) representing a variety of cancer diagnoses. RCT results included significantly decreased symptoms of stress, depression, anxiety, and self-regulatory fatigue, as well as improved health-related quality of life (HRQoL), when compared to participants in the usual-care control group (Borosund et al., 2022).

The current study engaged users of *StressProffen* during the RCT, employing qualitative methods to better understand potential mechanisms from the patient perspective when engaging with the intervention.

## Methods

### Study design and participants

This study reports on exploratory qualitative findings from semi-structured individual interviews related to use and potential benefit for a sub-group of cancer survivors who had access to the digital stress-management program *StressProffen* in a 12-month RCT (Borosund et al., 2020a; Borosund et al., 2022).

### StressProffen

**Figure 1:**
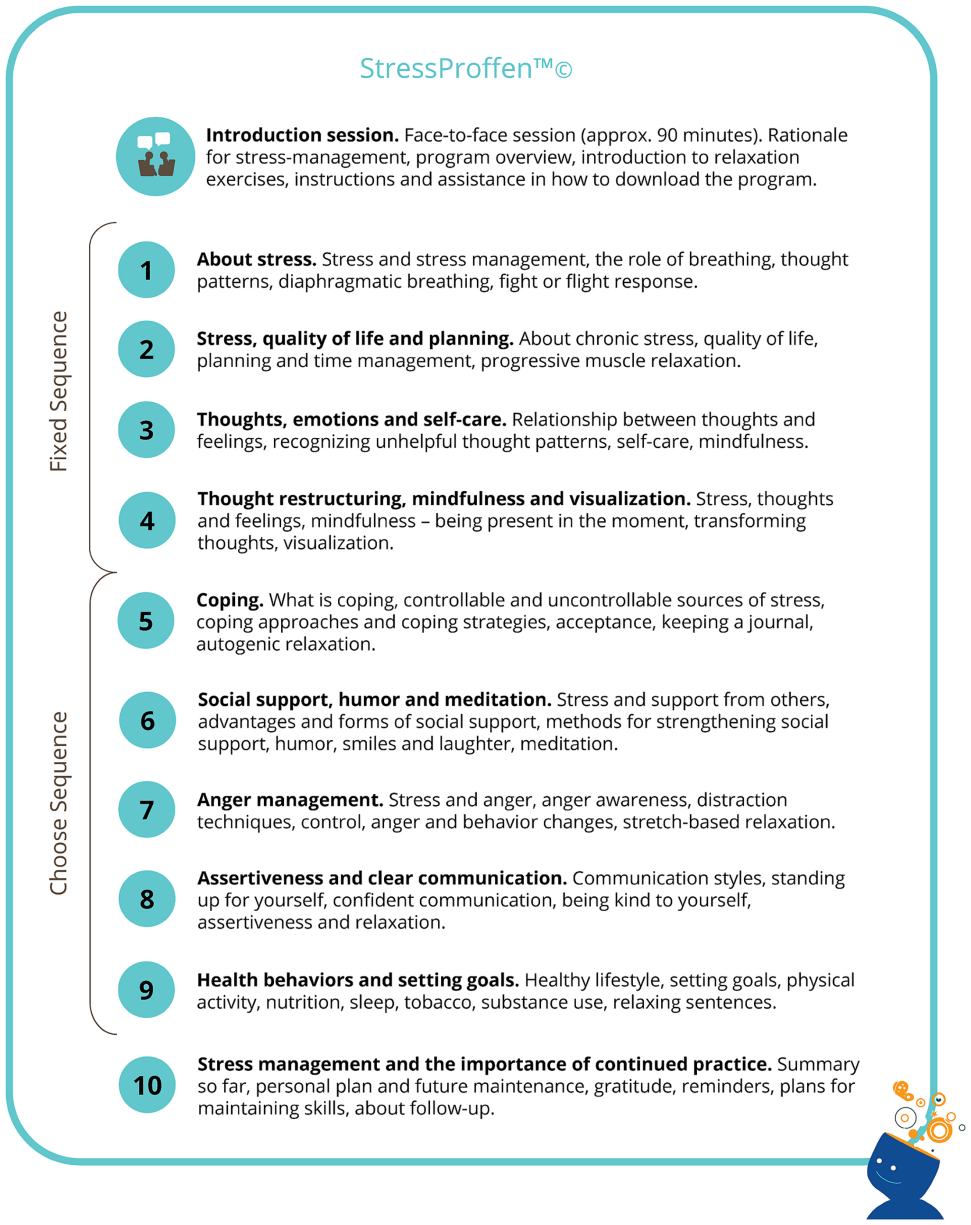
*StressProffen* program module content overview.

The digital stress-management intervention program *StressProffen* was designed and developed based on well-established cognitive-behavioral distress- and stress-management concepts (Andersen, 1992; Antoni et al., 2001; Antoni et al., 2023; Gudenkauf and Ehlers, 2018), with significant stakeholder (i.e., cancer survivors, health care providers, eHealth experts) involvement throughout (Borosund et al., 2018). The program consists of 10 primarily CBT-based modules, with content such as (1) stress; (2) stress, quality of life, and planning; (3) thoughts, emotions, and self-care; (4) thought restructuring, mindfulness, and visualization; (5) coping; (6) social support, humor, and meditation; (7) anger management; (8) assertiveness and clear communication; (9) health behaviors and setting goals; and (10) stress-management and the importance of continued practice. Please see Figure 1 for an overview of program content.

**Figure 2:**
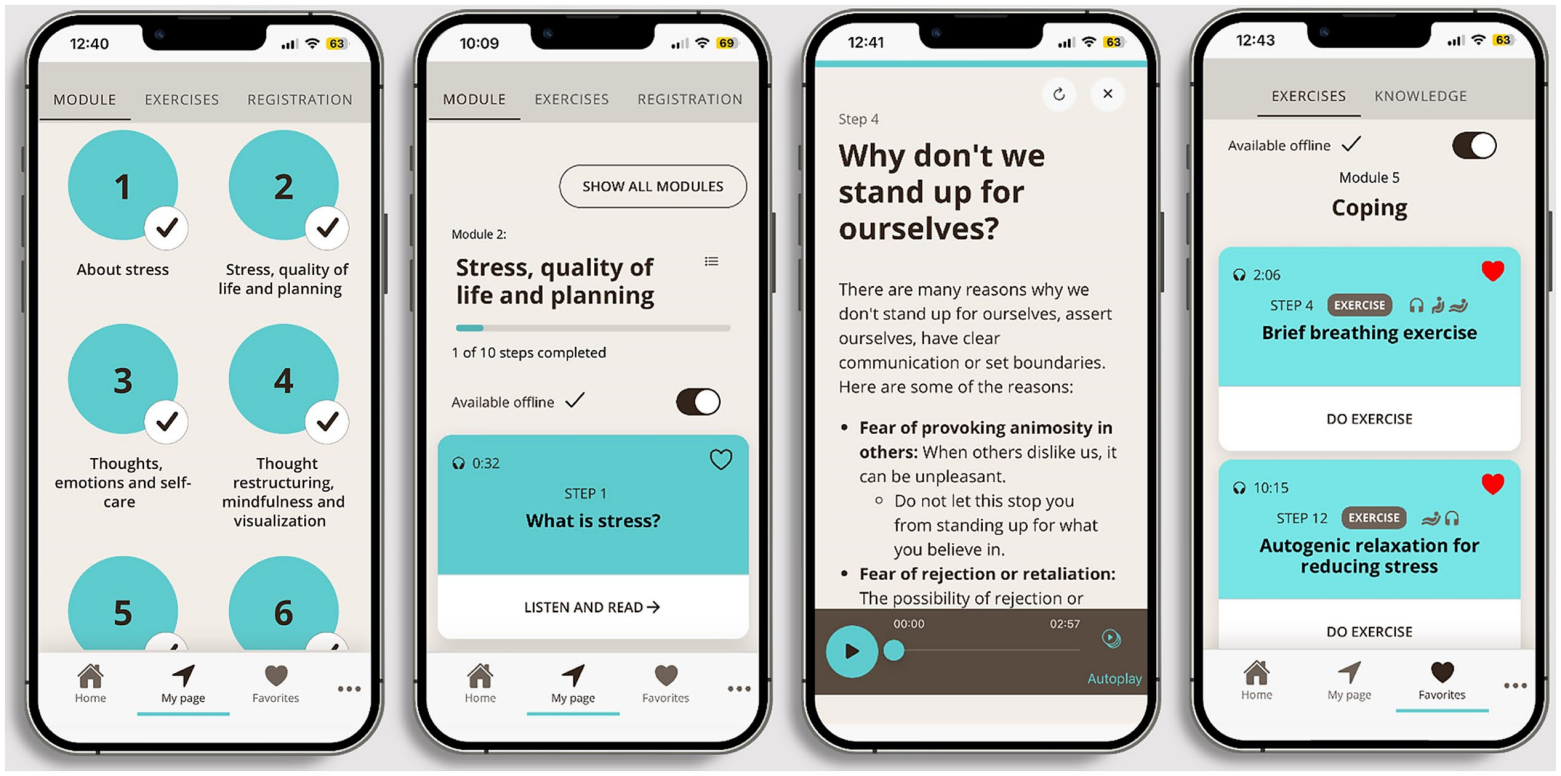
*StressProffen* example screenshots.

The first four modules are delivered in a fixed sequence, while the sequence of modules 5–9 can be individually chosen to allow for personalization. Each module contains a combination of psychoeducational content and exercises (e.g., diaphragmatic breathing, progressive muscle relaxation, mindfulness, and visualization), and after completion of one module, a 3-day “practice time” is induced before the next module can be opened (Borosund et al., 2018; Borosund et al., 2020b). Users can choose between reading and listening when using the program, and options to choose parts of the program as “favorites” allow for further personalization. Such favorites can then easily be re-used through a personal “My page” section, as can details related to program progress. Please see Figure 2 for *StressProffen* screenshot examples.

To further encourage engagement and individual follow-up, the *StressProffen* digital intervention program was delivered in a simple blended-care model with an in-person introduction session and two follow-up telephone calls at approximately 2–3 weeks and 6–7 weeks post the introduction session. Study personnel involved in the blended care model were trained by the principal investigator (PI; LSN), a doctoral-level licensed clinical health psychologist. The structured introduction session introduced participants to well-established stress-management concepts, provided help downloading *StressProffen*, and included guided practice on how to use the program. The follow-up telephone calls were semi-structured and conducted to see how participants were doing and inquire about whether they had any questions about the use of *StressProffen*. Participants were also informed that they could contact the study staff through a project telephone number on weekdays (i.e., between 9 a.m. and 3 p.m.) in the case of technical difficulties or related questions.

### Recruitment and data collection

Inclusion criteria for the RCT (*n* = 172) involved any type and stage of cancer, currently or recently in cancer treatment (maximum 1 year since completion of cancer treatment); ≥18 years old; able to speak, read, and understand Norwegian; having access to smartphone or tablet; and being able to attend an in-person *StressProffen* introduction session (Borosund et al., 2022). A detailed description of RCT study procedures can be seen in the original *StressProffen* RCT publications (Borosund et al., 2020a; Borosund et al., 2022).

Upon study enrollment into the RCT, participants were asked whether they could be contacted about participating in potential individual interviews post study completion (i.e., after completing 12-month outcome measures). To ensure heterogeneous representation, a matrix was created related to age (i.e., seeking a broad age spectrum), sex (male, female), diagnosis (i.e., breast cancer or other diagnosis), and program progression after 12 months (i.e., progress status 1–2 modules; 3–6 modules; or 7–10 modules). Based on the matrix, a selection of 39 participants in the intervention group (i.e., *n* = 61, having had access to *StressProffen* for 12 months and completed 12-month outcome measures) were invited, through text messages or telephone calls, to participate in the qualitative interviews. Study personnel were trained by the PI (LSN), and they conducted the interviews by telephone, using a semi-structured interview guide exploring topics such as general impressions, program use, lessons learned, what participants found useful (or not), and how these factors contributed to change (e.g., improved functioning). The interviews lasted from 12 to 58 min (median 25 min) and were audio-recorded and transcribed verbatim.

### Data analysis

Interview transcripts were analyzed based on the principles of thematic analysis as described by Braun and Clarke (2006). Thematic analysis was deemed a suitable analytical approach for this study, as the focus was on exploring patients’ experiences with using *StressProffen*, particularly in relation to psychological and behavioral change, rather than the subjective experience of stress-management as a phenomenon. The first author (CV) read through the transcripts and took notes to become familiar with the content. Using an inductive approach, the first author then analyzed the transcripts into codes as they emerged in the transcripts, aiming to explore the patients’ perspectives on *StressProffen* use and potential benefit, particularly how *StressProffen* had contributed to psychological and behavioral change in the participants’ lives. The second author (EB) independently coded a random selection of five interview transcripts, and the first and second authors then developed the coding themes and the interpretation of the results. Each theme was then re-examined, identifying variations and similarities within the themes and associated codes. The first, second, and senior authors (i.e., CV, EB, LSN) then discussed and reviewed the themes and codes, subsequently renaming and re-arranging the themes into a final structure. The three authors involved in the analysis held numerous meetings throughout the process, involving either two or all three of them. Consensus was reached through discussions, where the original transcripts were consulted in cases of uncertainty. There was, however, relatively little uncertainty regarding the interpretation of the participants’ perspectives, as the authors analyzing the transcripts had extensive experience working with the *StressProffen* intervention over a long period of time and across multiple sub-studies. Please see Appendix I for more details about themes and corresponding codes.

### Ethics

The study was approved by institutional research review board equivalents, including the Regional Committee for Medical and Health Research Ethics (approval number: 2016/14369) and the Hospital Privacy Protection Committee (approval number: 2015/10204). The RCT study was registered in ClinicalTrials (Clinicaltrials.gov: NCT02939612) prior to enrollment of participants. All participants provided written informed consent.

## Results

### Participant information

Of the 39 participants invited to participate in interviews, 26 (67%) agreed to participate. Participants (*N* = 26) were median of 53 years old, and 81% were women. Participant demographics are provided in Table 1.

**Table 1:** Baseline socio-demographic and disease-related measures (*N* = 26).

Characteristics	Participants (*N* = 26)	
Age (years), median (range)	53	(24–74)
Sex, *n* (%)		
Female	21	(81)
Male	5	(19)
Marital status, *n* (%)		
Married/cohabitating	16	(62)
Single/divorced	10	(38)
Education, *n* (%)		
Elementary/high school	7	(27)
University/college ≤4 years	8	(31)
University/college >4 years	11	(42)
Employment status, *n* (%)		
Full-time/part-time work	5	(19)
Sick leave/disability benefits	19	(73)
Retired/other	2	(8)
Diagnosis, *n* (%)		
Breast cancer	11	(42)
Lymphoma	3	(11)
Melanoma	2	(8)
Colon cancer	2	(8)
Other^a^	8	(31)
Metastases, *n* (%)	4	(15)
Months since diagnosis, median (range)	5	(0.25–84.0)
Program progression after 12 months, *n* (%)		
1–2 modules	1	(4)
3–6 modules	5	(19)
7–10 modules	20	(77)

^a^Other includes prostate cancer, brain cancer, cervical cancer, ovarian cancer, sarcoma, bladder cancer, head and neck cancer, and bladder cancer.

### Overview

**Figure 3:**
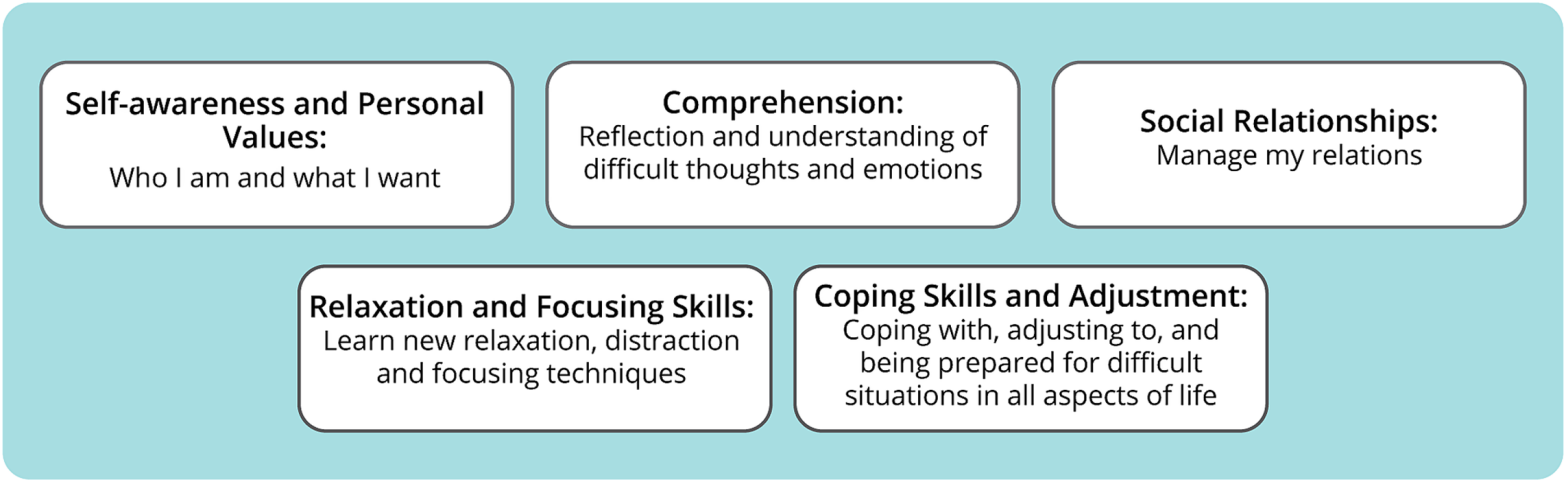
Overview of change-related themes after 12 months access to *StressProffen*.

Transcripts from the interviews were analyzed, and five themes were identified describing how *StressProffen* had contributed to changes in the participants’ lives, including: (1) *Self-awareness and personal values*; who I am and what I want, (2) *Comprehension*; reflection and understanding of difficult thoughts and emotions, (3) *Social relationships*; manage my relations, (4) *Relaxation and focusing skills;* Learn new relaxation, distraction, and focusing techniques; and (5) *Coping skills and adjustment;* coping with, adjusting to, and being prepared for difficult situations in all aspects of life (i.e., not only related to cancer). Figure 3 provides an illustration of these themes.

### Self-awareness and personal values: who I am and what I want

Participants described how *StressProffen* had helped them become more aware of aspects related to the philosophical questions of “Who am I” and “What do I want in life,” indicating a strengthening of meaning and purpose in life. They also identified *StressProffen* as having helped them retain their sense of self and maintain some degree of normality and positivity in life. Explaining how it had been crucial for them not to lose their composure during challenging times in the cancer journey, the participants stated that through learning to set their own personal goals, they were able to take greater responsibility for their own situation. One participant compared the situation to constructing a house from the ground up:

*There was kind of a longing inside me I think, to get my life back on track after the cancer diagnosis. I had to embrace the things that were good in my life already. And in a way, I built my house with a new foundation and a new base […] And that's where this app [StressProffen] became a great building block in my new house, a foundation I could fully rely on, where I could further develop everything I already had going for me, incorporating it with all the new things I gained through the app, becoming a newer, better version of myself. As a cancer patient and cancer survivor. And I thought that was wonderful. (Study participant #177)*

Participants also reported having put significant effort into getting to know themselves better through the *StressProffen* program, and as a result had achieved a better awareness of their own self, emotions, and values. One participant, for example, described how using *StressProffen* had facilitated a profound connection with their inner self:

*I get that deep inner connection with myself. […] And I feel that it happens for about two to four minutes, then I feel it very much, I am sort of inside myself, in what some would call a more meditative state (alpha) or something like that. (#67)*

### Comprehension: reflection and understanding of difficult thoughts and emotions

Participants consistently conveyed having experienced and struggled with anxiety, in particular with health-related anxiety, worry, and rumination, concerns about their future and what tomorrow might bring, as well as mortality concerns (i.e., fear of death and dying). They described *StressProffen* as assisting them in contemplating these types of thoughts and emotions, aiding them in organizing and managing the thoughts and feelings associated with the cancer journey. As one of the participants said:

*The motivation [for using StressProffen] was that I was very focused on not letting fear or anxiety, and such, control my life. (#159)*

Participants also referred to *StressProffen* as providing them with a better understanding of the connection between thoughts, feelings, and reactions, and described how this motivated them to attempt to change their mindset to embrace a more positive attitude now, and to let negative thoughts pass without dwelling on them too much. They reported having realized how thoughts are not inherently dangerous, real, or absolute truths, but simply thoughts. As stated by one of the participants:

*What has been useful is what has been said in the various modules; understanding the reasons behind my reactions, why I have them, so I can go back and understand why I have those exact feelings, thoughts, and all that. I think that has been really nice. (#180)*

Participants also described having learned how to distinguish between thoughts they could do something about and those they could not, having become more conscious of where to direct their efforts and energy.

### Social relationships: manage my relations better

Several of the participants described having experienced changes in their relationships with family and friends when being diagnosed with cancer, describing these changes as difficult to encounter and deal with. They described *StressProffen* as having provided them with suggestions for clear communication in such situations, subsequently contributing to better communication with their family and friends. One of the participants even mentioned how *StressProffen* had been helpful during the demanding process of ending the relationship with their partner, and several participants shared how they had learned to prioritize self-care and their own needs, becoming more assertive in the process. As one participant stated:

*So, I have used it [StressProffen] to help stand up for myself, and to communicate clearly and such, as those are things I have found to be very difficult. (#85)*

Some of the participants also described *StressProffen* as having helped them evaluate and assess their support network, contemplating which relationships they found energizing and which were draining. As reported by one of the participants:

*Taking some time to consider current relationships, reflecting on which ones provide energy and which are draining. It has been beneficial for me. (#70)*

### Relaxation and focusing skills: learn new relaxation, distraction, and focusing techniques

Most of the participants reported having found the *StressProffen* stress-management techniques and skills exercises, particularly the breathing exercises, beneficial. Participants shared how they had learned new breathing techniques that helped them calm down, regain control of their thoughts and feelings, and physically lower their shoulders and basically breathe using their abdomen. One participant even identified the diaphragmatic breathing exercises as the most crucial feature of *StressProffen* for them. Many described focusing on their breath and mastering diaphragmatic breathing as being a new experience, for example, as stated by one of the participants:

*Before, I think I tended to have more shallow breathing, particularly when I had a lot going on. And when I started using StressProffen, I noticed that I was mostly breathing from the upper part of my chest almost all the time when I had a lot on my plate. It can still happen, but now I recognize it quickly, and I can sense what is about to happen and consciously take deep abdominal breaths, filling my belly [with air] and then holding my breath slightly before exhaling again. (#163)*

Many of the participants also described the mindfulness exercises as beneficial, having helped them become more relaxed, increasing attention to the present moment, and ultimately leading to a greater sense of real-time presence in life (i.e., as opposed to the past or the future). The visualization techniques were also reported as useful to many, whether for calming down or as a preparation method for challenging tasks. As one participant stated:

*The (visualization) exercises have really helped me a lot. When I find myself in a situation or caught up in a lot of thoughts about hospital appointments and such, I use them automatically now. (#177)*

A number of participants also reported having used *StressProffen* when experiencing sleep difficulties, stating that the relaxation exercises help them quiet down before going to bed. One participant said:

*I lay there listening to it [StressProffen] and fell asleep every time, you know, breathing and everything — visualizing and all that stuff. (#105)*

Another participant highlighted how *StressProffen* was helpful when waking up during the night, stating:

*I sometimes slept poorly, and then I could open the app and use some of those exercises to try to fall asleep, especially in the middle of the night when you wake up and cannot sleep. I have actually used it quite a lot for help with sleep. In certain periods, I've been like that, sleeping poorly, typically being unable to sleep, and then there's the choice of using it [StressProffen] as opposed to taking sleeping medication. (#140)*

### Coping skills and adjustment: coping with, adjusting to, and being better prepared for difficult situations in all aspects of life

*StressProffen* was described as having played a significant role in the lives of many participants. Having gone through difficult times during their cancer journey, many participants stated that *StressProffen* should be offered to cancer patients at the time of their diagnosis to help them prepare and cope with what was ongoing on and what was to come. As one participant described:

*I think it [StressProffen] was very, very helpful. It was the only thing I had. […] If I hadn't had it, I would still just be lying there crying. […] And if I didn't have that app at that time, I think I would have lost it. (#105)*

While some participants described having gradually returned to a sense of normalcy after their cancer diagnosis and treatment, others reported experiencing continued struggles with adjusting to life after treatment, including new daily routines and their quality of life. Participants reported being able to use the knowledge gained from *StressProffen* when facing challenging situations related to their cancer, for example, when feeling sick during and after chemotherapy, or when experiencing later side effects such as fatigue, or before having a follow-up consultation at the hospital. As one participant stated:

*When I'm about to go in for a check-up, and as I get closer to the hospital, I know I'll be heading into a challenging situation, and I feel my body becoming stressed. And then I’ve used that app. (#163)*

Participants also described having benefited from using *StressProffen* in other parts of life (i.e., not only related to cancer), for example, having used *StressProffen* when feeling stressed during daily activities, or when just feeling overwhelmed with daily events. Depicting knowledge about which program sections to access and listen to in such situations, participants referred to *StressProffen* as providing instant guidance. The *StressProffen* program had reportedly also proven useful when facing stressful situations at work. As one participant said:

*I have benefited greatly from it [StressProffen]. Especially when there have been things with work and such. You know, work is my stressor, causing my level of stress. (#161)*

Many of the participants expressed gratitude for having access to *StressProffen* and suggested that not only cancer patients but anyone could benefit from using *StressProffen.* As declared by one of the participants:

*Get it [StressProffen] out to the public. You have put so much work into this, and done such a great job with it, it is about time it gets out there and gets used. I believe it can help many. Well, you won’t be cured by it, but you'll actually get better if you can lower your own pulse, gaining peace of mind. (#61)*

## Discussion

This qualitative study identified five themes advancing current understanding of potential mechanisms of cancer stress-management interventions that are built upon a foundation of the CBT framework. In explaining why they found *StressProffen* useful and helpful, participants identified mechanisms of improved *self-awareness and personal values* about “who I am and what I want” (i.e., meaning, purpose, and values), improved *comprehension* in terms of reflection and understanding of difficult thoughts and emotions, increased ability to manage *social relationships,* enhanced *relaxation and focusing skills*, and improved *coping skills and adjustment*, as in coping with, adjusting to, and being prepared for difficult situations in all aspects of life, including the cancer journey from diagnosis to post-treatment. All five themes identified are in line with existing research pointing to positive impact from CBT interventions in cancer, including enhanced self-awareness and comprehension, improved social interactions and relationships, strengthened skills for relaxation and focus, and improved coping, adjustment, and preparedness (Antoni et al., 2023; Savaş, 2025).

### Theme 1: self-awareness and personal values

Describing having access to *StressProffen* as improving *self-awareness*, participants reported having become more aware of their own identity, who they were, and what they valued, and also more aware of patterns within their own reactions. Participants also described learning how to set goals in line with their own values, realizing the importance of taking more responsibility for their own day-to-day life, which underlines the concept of setting achievable goals and monitoring progress toward skills development in CBT, supporting motivation and the likelihood for goals achievement (Dobson and Dobson, 2018). Recognizing the importance of identifying and embracing the good things in life, not just focusing on difficult or challenging aspects, the participants also described having become “better versions” of themselves. Consistent with research showing how CBT interventions can help foster adaptive coping through increased self-awareness (Savaş, 2025), the participants also described learning how to keep their composure even during difficult times and becoming more aware of ways to challenge and improve their own individual approach to life.

### Theme 2: comprehension

In accordance with existing research (Antoni and Dhabhar, 2019; Andersen et al., 1994; Grassi et al., 2017; Singer, 2018; Stanton et al., 2015; Stanton, 2006; Stanton, 2012), participants reported having experienced difficult thoughts and emotions as a consequence of their cancer diagnosis, treatment, and potential future impact on their health and wellbeing. They described improved *comprehension* of the relationship between thoughts, emotions, and behavior; portrayed enhanced ability to recognize unhelpful thought patterns; and learning to contemplate, organize, and manage thoughts and emotions better as a consequence of this skill development. This reflects the core essence of CBT, recognizing the connection between thoughts, emotions, and behaviors, and using this comprehension to improve wellbeing and coping (Beck, 2011; Beck, 2020; Dobson and Dozois, 2010; Dobson and Dobson, 2018).

In accordance with the premise that CBT can help people identify, challenge, and modify negative thought patterns, participants explained how understanding the connection between their thoughts, emotions, and behavior had helped them recognize which thoughts were not helpful, and to prioritize things they could control, rather than dwell on things that could not be changed. Interviewed participants also described how learned skills, such as mindfulness, helped them stay in the moment without ruminating too much, or at least recognizing the rumination or thought processes for what they were, and then being able to redirect to less intrusive, more constructive thoughts.

### Theme 3: social relationships

Having experienced challenging changes in relationships to significant others, family, and friends following cancer diagnosis and treatment, participants also described improved problem-solving and communications within *social relationships* through the use of *StressProffen*. They reported having learned to communicate more clearly with those around them, having realized the need and how to become more assertive and prioritize self-care. This supports the notion of CBT incorporating social skills training, including how to communicate clearly and assertively, building competency and confidence, and fostering mutual understanding, social skills, and social connections (Dobson and Dobson, 2018). Through becoming more aware of their own social support network, participants also described having learned to recognize which relationships were energizing, how to prioritize those relationships, and how to identify and set boundaries for more draining relationships.

### Theme 4: relaxation and focusing skills

CBT-based stress-management interventions often include a variety of participatory experiences and skill exercises, including but not limited to *relaxation skills* (e.g., diaphragmatic breathing and progressive muscle relaxation), distraction (e.g., counting), and *focusing* (e.g., mindfulness and meditation) skills (Antoni et al., 2023). Participants in the current study referred to the breathing exercises in *StressProffen* as particularly beneficial, describing how deep, diaphragmatic breathing could help them induce relaxation and calmness in a new and powerful way. This is also in line with RCT findings showing diaphragmatic breathing and progressive muscle relaxation, with deep breathing as the most used exercise when measured at 3 months (Borosund et al., 2020a).

Participants described using the relaxation exercises when struggling to fall asleep, or when waking up and having a difficult time going back to sleep during the night, which is in line with existing research showing positive impact from cognitive behavioral stress-management and relaxation interventions on sleep quality (Murawski et al., 2018). Some participants even reported replacing sleep medication with these types of relaxation skills from *StressProffen*. Other types of skills described as beneficial included guided imagery exercises, referred to as particularly useful for distraction and inducing a sense of calmness, and mindfulness exercises, aiding with being more present and aware in day-to-day settings. Practicing the exercises found in *StressProffen* was depicted as vital for obtaining effect, though, with participants stressing the need for consistent practice in order to achieve more automatic application, eventually without even opening the app.

### Theme 5: coping skills and adjustment

Access to *StressProffen* was referred to by participants as facilitating *coping* skills and *adjusting* to life after diagnosis and treatment. Participants described receiving guidance; learning new approaches for stress-management; and using the knowledge gained to adjust to the new situation, be more prepared, and be able to cope with challenges better and also when feeling overwhelmed. Some even referred to *StressProffen* as pivotal, a glimmer of hope to cling to during the darkest, most difficult, scary, or nauseating times. While describing recommendations for *StressProffen* to be distributed at an early stage in the cancer trajectory, participants underlined the importance of later stage access as well, for example, when treatment was completed and people often expected the cancer survivors to “be back to normal,” even though their lives might feel forever changed, consistent with prior reports (Ehlers et al., 2023).

The behavioral activation and problem-solving aspects of CBT (Beck, 2011; Beck, 2020; Dobson and Dozois, 2010; Dobson and Dobson, 2018) can likely be of benefit regardless of illness or mental challenges, and participants described S*tressProffen* beneficial also outside of dealing with cancer, for example, when feeling overwhelmed with activities and obligations in general, describing taking breaks and listening to *StressProffen* as re-energizing in a hectic day-to-day life. Participants even expressed being grateful for having been given access to the digital intervention, stating that anyone could likely benefit from use at some level, findings also noted by participants in the *StressProffen* feasibility pilot study (Borosund et al., 2020b).

Facilitating improved coping skills, preparedness, and adjustment to adverse challenges, CBT-based interventions such as *StressProffen* may also facilitate resilience (i.e., ability to adjust and recover from adversity) (Matheus Pinto et al., 2024), as well as contribute to better self-regulation (i.e., capacity to regulate thoughts, emotions, and behavior) in the face of challenges (Solberg Nes et al., 2014; McGonigal, 2013), as also seen in the quantitative RCT findings (Borosund et al., 2022). The current findings underline this notion through participants, pointing to improved understanding and subsequent ability to persist and manage challenges, as well as enhanced skills and subsequent capacity for coping, adjustment, and control.

### Strengths and limitations

The current study has a number of strengths, including the systematic matrix-based recruitment strategy allowing for a diverse sample of intervention group participants (i.e., age, sex, cancer diagnosis, and program progression). The interviews were also conducted with approximately 43% of the intervention group participants completing the 12-month RCT outcome measures (*n* = 61; Borosund et al., 2022), allowing for rich, qualitative results to complement quantitative RCT results (Borosund et al., 2022; Borosund et al., 2020a), and in turn enriching the interpretation of post 12-month intervention findings (O'Cathain et al., 2013; Richards et al., 2019).

The study also has some limitations that should be recognized. First, while participants were not required to complete all sessions of the intervention, they did have to complete the 12-month outcome measures, potentially already indicating study engagement. Other perspectives might have been expressed by those not completing the study measures. Also, those agreeing to be interviewed could likely be assumed to be motivated to share their experience, and even though participants with low progression rates were invited for interviews to strive for a rich and profound picture of program use and perceived benefits, most agreeing to be interviewed had completed 7–10 modules. This could suggest a possible self-selection bias, with more engaged users being overrepresented in the study, which may have influenced the findings. All of these factors might limit the transferability of findings to other settings or other diagnoses.

Second, the majority of participants interviewed were women (81%), which introduces another transferability-related issue. Female/male specific response patterns were, however, not detected in the qualitative data obtained, and distribution does reflect the percentages seen in the RCT (i.e., 82% women (Borosund et al., 2022)). Also, a majority of female participants appears to be common for these types of studies, perhaps reflecting a basic sex difference in motivation for self-management interventions, and motivation for engagement is after all vital for intervention benefit (Gan et al., 2021; Lipschitz et al., 2022; Meyerowitz-Katz et al., 2020).

Third, the education level was high among the participants in this study, with 73% reporting having university or college-level education. This could be one reason why the participants found the CBT-based *StressProffen* intervention to be so helpful, supporting existing research showing how education levels can increase intervention efficacy in terms of psychological outcomes (Low et al., 2024), and how people with higher education may benefit more from CBT-type approaches to self-management(Bosma et al., 2011). Education level has also been associated with health literacy (WHO, 2024), and suggestions have been made that low health literacy may impact necessary activities for capacity for self-management (Mackey et al., 2016). It should, however, be noted that although the education level was high in the current study sample, an even larger percentage of participants with higher education was reported in the RCT [i.e., 81% having university or college level education (Borosund et al., 2022)].

Finally, while the study included cancer survivors with a wide range of cancer diagnoses for applicability to real-world healthcare, the majority (42%) were diagnosed with breast cancer. It is possible that more options for tailoring and individualization (Klooster et al., 2024; Lee et al., 2025), for example, based on specific cancer diagnoses, stages, or other conditions, could have contributed to bolstering the effect and sense of impact from *StressProffen* even further.

### Future directions

The current study shows how a mixed methods approach can contribute to more in-depth interpretation and comprehension of large-scale RCT outcomes and specifically therapeutic mechanisms of significant trial outcomes. Future research should hence consider employing mixed methods approaches when seeking to explore the impact of health interventions. With healthcare services worldwide under pressure, digital health interventions have the potential to improve access and impact for cancer survivors and patient populations in general. This study shows how a CBT-based digital stress-management intervention can help improve psychosocial wellbeing for cancer survivors, and which factors contribute specifically and how. Based on this type of in-depth comprehension of what might contribute to individual improvement, future research should consider these themes to further tailor and individualize digital health interventions in support of cancer survivors.

In terms of practical recommendations for the implementation of evidence-based digital stress-management interventions such as *StressProffen*, healthcare and community providers should consider some form of introduction (e.g., with examples of potential benefits) and/or simple, individual, follow-up mechanisms for users, as even simple forms of blended care appear beneficial to encourage intervention engagement, use, and hence effect.

## Conclusion

The current qualitative study showed how cancer survivors who had access to the *StressProffen* digital health intervention program for 12 months experienced positive changes in terms of enhanced self-awareness, comprehension in terms of reflection and understanding of difficult thoughts and emotions, improved ways to deal with social relations, learning, and engaging in relaxation and focusing techniques, and improving ways to cope with, adjust to, and be prepared for difficult situations in all aspects of life. Findings help explain how patients viewed their individual progress, resulting in improved quantitative trial outcomes (i.e., decreased stress, anxiety, and depression, as well as improved self-regulatory capacity and QoL for cancer survivors after 12 months *StressProffen* intervention access). Additionally, utilization of a mixed methods approach, including quantitative and qualitative analyses, can contribute to a better understanding of intervention mechanisms through the patient user perspective.

## Data Availability

The datasets presented in this article are not readily available because data sets from this study are, due to the nature of patient-sensitive information, not available for public sharing through public archives or repositories. However, deidentified data from this study will be made available in accordance with institutional standards upon contacting the corresponding author. Requests to access the datasets should be directed to Lise Solberg Nes, solbli@ous-hf.no.
