# Supplementary material for: Potential mechanisms within the digital stress-management intervention StressProffen for cancer survivors: a qualitative study post randomized controlled trial
Source: Front Psychol. 2026 Jan 12;16:1703540. doi: 10.3389/fpsyg.2025.1703540 (PMC12833618; doi:10.3389/fpsyg.2025.1703540)
Supplement: Supplementary file 1 [file Supplementary_file_1.docx]

**Appendix I - Supplementary material:**

**Themes and corresponding codes – StressProffen 12-month interviews**

- **(translated from original thematic analyses coding in Norwegian)**

### Theme 1: Self-awareness and Personal Values: Who I am and what I want

- Get to know my inner self
- What do I want
- Learn to set goals
- Take responsibility
- Maintain composure

### Theme 2: Comprehension: Reflection and understanding of difficult thoughts and emotions

- Anxiety
- Fear
- Rumination
- Concerns
- Connection between thoughts, feelings and reactions

### Theme 3: Social Relationships: Manage my relations better

- Social support
- Relationships
- Communication
- Assertiveness

### Theme 4: Relaxation and Focusing Skills: Learn new relaxation, distraction and focusing techniques

- Breathing techniques
- Mindfulness
- Meditation
- Visualization
- Sleep-related exercises

### Theme 5: Coping Skills and Adjustment: Coping with, adjusting to, and being better prepared for difficult situations in all aspects of life

- Stress in day-to-day life
- Work challenges
- Body/mind balance/imbalance
- Cancer-related symptoms during and after treatment
- Treatment side effects/preparations
